# HIV prevalence and factors associated with HIV positivity among Black people in primary care in Porto Alegre, Brazil, 2020-2022: a cross-sectional study

**DOI:** 10.1590/S2237-96222025v34e20240014.en

**Published:** 2025-05-12

**Authors:** Emerson Silveira de Brito, Marsam Alves de Teixeira, Rafael Steffens Martins, Ben Hur Graboski Pinheiro, Ana Carolina Monteiro da Rocha, Cáren Nunes de Oliveira, Thayane Fraga de Paula, Thayane Martins Dornelles

**Affiliations:** 1 Universidade Federal de Ciências da Saúde, Programa de Pós-graduação em Ciências da Saúde, Porto Alegre, RS, Brazil; 2 Afya Educação Médica, Diretoria de Pós-Graduação Médica, Porto Alegre, RS, Brazil; 3 Associação Hospitalar Moinhos de Vento, Porto Alegre, RS, Brazil; 4 Instituto René Rachou, Fiocruz Minas Gerais, Belo Horizonte, MG, Brazil

**Keywords:** Sexually Transmitted Diseases, HIV Infections, Black People, Health of Ethnic Minorities, Cross-Sectional Studies, Enfermedades de Transmisión Sexual, Infecciones por VIH, Población Negra, Salud de las Minorías Étnicas, Estudios Transversales

## Abstract

**Objective:**

To examine HIV prevalence among primary care service users and to investigate factors associated with positive HIV test results among Black people.

**Methods:**

This was a cross-sectional study with data from rapid HIV testing performed in health centers in Porto Alegre-RS, Brazil. Sociodemographic differences according to race/skin color and HIV test result were analyzed using the Chi-square test and Poisson regression with robust variance.

**Results:**

Out of 92,345 people tested, 38% were Black, with 3.4% HIV prevalence. Among Black people the following were associated with higher HIV prevalence ratios (PR): being male (PR 1.62; 95% confidence interval [95%CI] 1.41; 1.85), having elementary education (PR 1.69; 95%CI 1.27; 2.24), having tuberculosis (PR 1.76; 95%CI 1.22; 2.54) and being a street dweller (PR 1.75; 95%CI 1.41; 2.18).

**Conclusion:**

Black people, especially Black men with lower education levels, tuberculosis and street dwellers, have higher HIV prevalence, requiring greater attention from prevention strategies and testing for sexually transmitted infections.

## Resumen

**Objetivo:**

Verificar la prevalencia del VIH en usuarios de atención primaria e investigar los factores asociados con la prueba positiva del VIH en personas de raza negra.

**Métodos:**

Estudio transversal con datos de pruebas rápidas de VIH en unidades de salud de Porto Alegre. Las diferencias sociodemográficas según raza/color de piel y resultado de la prueba de VIH se analizaron mediante la prueba de Chi-cuadrado y regresión de Poisson con varianza robusta.

**Resultados:**

Entre 92.345 personas analizadas, el 38% eran negros, con una prevalencia del VIH del 3,4%. Entre la población negra, se asoció con una mayor razón de prevalencia (RP) de VIH: ser hombre (RP 1,62; intervalo de confianza del 95% [IC95%] 1,41; 1,85), tener educación primaria (RP 1,69; IC95% 1,27; 2,24), tener tuberculosis (RP 1,76; IC95% 1,22; 2,54) y ser habitante de la calle (RP). 1,75; IC 95%: 1,41; 2.18).

**Conclusión:**

La población negra, especialmente los hombres negros con menor educación, tuberculosis y habitantes de la calle, tienen una mayor prevalencia de VIH, lo que requiere mayor atención a las estrategias de prevención y pruebas de infecciones de transmisión sexual.

## Introduction

The human immunodeficiency virus (HIV) is a major public health challenge due to the magnitude, complexity and difficulty in controlling this epidemic in society (1). It is estimated that 39 million individuals are infected with the virus worldwide, and even with progress with management of this infection, it remains a latent problem, especially when assessing disproportionate differences in HIV prevalence in more vulnerable populations, such as Black people (2-4).

In Brazil, 37,023 new HIV infection cases were reported in 2020 and 43,403 in 2022, representing an increase of 17.2% (5). Ministry of Health data that assessed these new diagnoses according to race/skin color indicate an increase in diagnoses among people of Black and mixed race/skin color and a decrease in new cases among White people. In 2023, Black people accounted for 61.6% of new cases (49.3% mixed race and 12.2% Black), while White people accounted for 32.9% (5-6). In the same year, 63% of deaths due to AIDS in Brazil occurred among Black people (5). Data from other countries, such as the United States, show that 31% of all cases of chlamydia, gonorrhea, and syphilis occurred among Black people in 2021, even though they only accounted for approximately 12% of that country’s population (6).

In the quest to understand these inequities, studies show that Black people suffer greater restrictions in accessing health services, and when Black people are treated, they receive poorer quality care and less effective solutions (7). When compared to White people, the social inequalities faced by people of Black and mixed race/skin color result in higher incidence of socially determined diseases, in addition to higher mortality rates, due to health policy neglect (8-9), which can be explained by a complex set of different historically determined types of vulnerabilities (social, individual and structural) (10-11).

Given this epidemiological scenario among Black people, it is crucial to produce new regional estimates of HIV prevalence and to conduct in-depth analysis of factors associated with higher HIV detection in this population. These initiatives are essential for strengthening health policies and developing specific strategies aimed at the most vulnerable groups. In this context, the objective of this study was to examine HIV prevalence among primary care service users and to investigate factors associated with positive HIV test results among Black people.

## Methods

### Design

This is a cross-sectional study with people aged 12 years or older who underwent rapid HIV testing between January 2020 and November 2022 in primary health care services in Porto Alegre. We followed the Strengthening the Reporting of Observational Studies in Epidemiology guidelines when writing our report of this study (12).

### Participants

All individuals over 12 years of age were included in the study. Individuals for whom there was no date of birth were excluded from the database. The study data refer to individuals living in the city of Porto Alegre who underwent rapid HIV testing at the city’s health institutions, such as primary health care centers, in addition to the following specialized services: Specialized Care Services, Tuberculosis Treatment Reference Center and Psychosocial Care Center.

### Variables

Taking the data collected via Google Forms by the health services, we used the following variables: race/skin color (White, Black [Black and mixed race], Asian and Indigenous), sex (male, female), age (in years: 12-17, 18-25, 26-39, 40-59), year of rapid testing, education (elementary, high school, higher education), tuberculosis (no, yes), street dweller (no, yes). The result of the first HIV test (non-reactive, reactive and not performed) was also collected, followed by the confirmatory HIV test result in cases in which the first test was positive. Incomplete data due to missing answers and/or invalid answers were considered to be losses.

### Data source and measurement

The study was conducted using secondary data obtained from the Sexually Transmitted Infections Care Coordination Office in Porto Alegre. Information about each participant was recorded by professionals who perform rapid tests for sexually transmitted infections (STIs) completing a form via Google Forms, which is standard procedure in services that perform testing. Management of the variables contained on this form is carried out by the municipality’s STI technical team. The data were accessed from January 2023 onwards after being made available by the Sexually Transmitted Infections Care Coordination Office in Porto Alegre, where they were compiled on an Excel spreadsheet containing information from the municipality’s health services.

### Study size

The study consisted of a total of 99,641 participants who underwent rapid HIV testing in Porto Alegre. No sample size calculation was performed, as the study was based on all rapid testing data performed between January 2020 and November 2022.

### Statistical methods

The data were compiled in Excel spreadsheets and later coded for quantitative analysis using the Statistical Package for the Social Sciences (SPSS), version 29.

The sociodemographic profile of individuals who underwent rapid HIV testing was presented, broken down by race/skin color. Next, a comparison was made between sociodemographic profile according to rapid HIV test positivity between Black and White people. For both analyses, presented in Tables 1 and 2, the categorical variables were summarized using absolute frequencies and percentages, and differences between the groups were assessed using Pearson’s chi-square test.

In addition, univariate Poisson regression analysis with robust variance was performed for all variables of interest (model 1). For the multivariate analysis, a theoretical framework was structured in stages, based on the hierarchical model, to calculate associations between positive HIV test results (model outcome) and sociodemographic variables, seeking to identify, specifically among Black people, which characteristics could be independently associated with higher HIV prevalence. As such, the following models were used: model 1 – crude estimates (univariate model); model 2 – sex and education (elementary, high school or higher education); model 3 – sex, education and age group at the time of testing (12-17; 18-25; 26-39; 40-59 years); model 4 – sex, education, age group and history of tuberculosis (yes/no); and model 5 – sex, education, age group, history of tuberculosis and street dweller (yes/no).

## Results

We analyzed 99,641 STI rapid tests (syphilis, HIV, hepatitis B or C) performed during the period evaluated, of which 1,915 tests were excluded from the analyses due to lack of information on date of birth. Specifically in relation to HIV, 92,345 tests were performed and 2,319 had reactive results, resulting in a prevalence rate of 2.5% (95% confidence interval [95%CI] 2.4; 2.6). The mean and standard deviation of the age of the participants was 32.7±13.2 years. Table 1 presents the sociodemographic characteristics of the individuals who underwent rapid testing in public health services, showing that, among individuals with higher education, 73.8% were White and 25.4% were Black. Regarding HIV diagnoses, 52.4% of the reactive test results were for Black individuals. 

**Table 1:** Characteristics of individuals who underwent rapid HIV testing according to race/skin color classification. Porto Alegre, 2020-2022 (n=92,345)

Characteristics	Total	White n (%)	Black n (%)	Asian/ indigenous n (%)	p-value^a^
**Sex**					<0.001
Male	34,849	20614 (59.2)	13,854 (39.7)	381 (1.1)	
Female	57,496	35,231 (61.3)	21,713 (37.7)	552 (1.0)	
**Age group** (years)					
12-17	4,261	2,406 (56.5)	1,795 (42.1)	60 (1.4)	<0.001
18-25	29,023	16,914 (58.3)	11,817 (40.7)	292 (1.0)	
26-39	35,105	21,112 (60.1)	13,661 (39.0)	332 (0.9)	
40-59	17,407	10,955 (62.9)	6,260 (36.0)	192 (1.1)	
≥60	4,791	3,403 (71.0)	1,352 (28.2)	36 (0.8)	
Schooling					<0.001
Elementary	27,767	14,960 (52.9)	12,710 (45.8)	383 (1.3)	
High school	35,710	22,055 (61.8)	13,363 (37.4)	298 (0.8)	
Higher education	8,068	5,949 (73.8)	2,053 (25.4)	69 (0.8)	
Tuberculosis					<0.001
No	75,541	45,195 (59.9)	29,573 (39.1)	773 (1.0)	
Yes	725	381 (52.6)	338 (46.6)	6 (0.8)	
**Street dweller**					<0.001
No	74,022	44,479 (60.1)	28,801 (38.9)	742 (1.0)	
Yes	2,244	1,097 (48.9)	1,110 (49.5)	37 (1.6)	
**HIV RT result**					<0.001
Non-reactive	90,026	54,765 (60.8)	34,353 (38.2)	908 (1.0)	
Reactive	2,319	1,080 (46.5)	1,214 (52.4)	25 (1.1)	

Considering that the data was derived from a secondary questionnaire, there were losses in some variables, with the following proportions of valid responses: patient’s sex (98.7%), age (96.8%), education (76.4%), street dweller (81.5%), tuberculosis (81.5%) and race/skin color (98.7%). These variables were impacted by errors in questionnaire completion or questionnaire changes over time.

Table 2 shows that HIV prevalence among Black men was 4.7%, while among White men it was 2.9%. Among self-reported White and Black women, HIV prevalence was 1.4% and 2.6%, respectively. Considering Black people, a higher proportion of reactive HIV tests was identified in people aged 40 to 59 years (6.3%), with elementary education (4.2%), with tuberculosis (10.1%) and street dwellers (11.1%). When we analyzed these same variables for White people, we identified a higher prevalence of positive rapid tests among individuals aged 40 to 59 years (3.0%), with elementary education (2.8%), with tuberculosis (7.3%) and street dwellers (6.6%). 

**Table 2:** Participant characteristics and HIV rapid test positivity, by race/sin color. Porto Alegre, 2020-2022 (n=91.412)

	Black - n (%)			White - n (%)		
Characteristics	Non-reactive	Reactive	p-value^a^	Non-reactive	Reactive	p-value^a^
Sex			<0.001			<0.001
Male	13,204 (95.3)	650 (4.7)		20,016 (97.1)	598 (2.9)	
Female	21,149 (97.4)	564 (2.6)		34,749 (98.6)	482 (1.4)	
**Age group** (years)			<0.001			<0.001
12-17	1,776 (98.9)	19 (1.1)		2,389 (99.3)	17 (0.7)	
18-25	11,599 (98.2)	218 (1.8)		16,711 (98.8)	203 (1.2)	
26-39	13,137 (96.2)	524 (3.8)		20,660 (97.9)	452 (2.1)	
40-59	5,864 (93.7)	396 (6.3)		10,627 (97.0)	328 (3.0)	
≥60	1,314 (97.2)	38 (2.8)		3,349 (98.4)	54 (1.6)	
Schooling			<0.001			<0.001
Elementary	12,174 (95.8)	536 (4.2)		14,278 (97.2)	412 (2.8)	
High school	12,976 (97.1)	387 (2.9)		21,692 (98.4)	363 (1.6)	
Higher education	2,001 (97.5)	52 (2.5)		5,872 (98.7)	77 (1.3)	
Tuberculosis			<0.001			
No	28,570 (96.6)	1,003 (3.4)		44,324 (98.1)	871 (1.9)	
Yes	304 (89.9)	34 (10.1)		353 (92.7)	28 (7.3)	
**Street dweller**			<0.001			<0.001
No	27,887 (96.8)	914 (3.2)		43,652 (98.1)	181 (1.9)	
Yes	987 (88.9)	123 (11.1)		1,025 (86.4)	72 (6.6)	

The results of the multivariate analysis showed that the prevalence ratio among Black people with reactive rapid HIV test results was higher in men (PR 1.62; 95%CI 1.41; 1.85), people with elementary education (PR 1.69; 95%CI 1.27; 2.24), people with tuberculosis (PR 1.76; 95%CI 1.22; 2.54), and street dwellers (PR 1.75; 95%CI 1.41; 2.18) in the final model. It is noteworthy that Black people aged 40 to 59 years had a higher HIV infection prevalence ratio (PR 2.40; 95%CI 1.66; 3.47), while the 12-17 age group (PR 0.44; 95%CI 0.24; 0.82) was associated with the lowest prevalence (Table 3).

**Table 3:** Multivariate analysis of factors associated with HIV-reactive results among people of Black race/skin color. Porto Alegre, 2020-2022

Variable	Prevalence ratio (95% confidence interval [CI])				
	Model 1 (crude analysis)	Model 2^a^	Model 3^b^	Model 4^c^	Model 5^d^
Sex					
Male	1.84 (1.63; 2.09)	1.87 (1.65; 2.12)	1.74 (1.54; 1.98)	1.73 (1.52; 1.96)	1.62 (1.41; 1.85)
Female	1.00	1.00	1.00	1.00	1.00
Schooling					
Elementary	1.66 (1.25; 2.20)	1.69 (1.27; 2.23)	1.82 (1.37; 2.42)	1.30 (0.97; 1.74)	1.69 (1.27; 2.24)
High school	1.15 (0.87; 1.54)	1.15 (0.87; 1.53)	1.30 (0.97; 1.74)	1.80 (1.35; 2.39)	1.28 (0.96;1.71)
Higher education	1.00	1.00	1.00	1.00	1.00
**Age group** (years)					
12-17	0.38 (0.22; 0.66)		0.43 (0.23; 0.79)	0.44 (0.24; 0.81)	0.44 (0.24; 0.82)
18-25	0.67 (0.47; 0.94)		0.83 (0.57; 1.22)	0.84 (0.57; 1.23)	0.84 (0.57; 1.23)
26-39	1.38 (1.00; 1.91)		1.67 (1.17; 2.41)	1.68 (1.16; 2.42)	1.63 (1.13; 2.36)
40-59	2.24 (1.61; 3.11)		2.54 (1.77; 3.67)	2.55 (1.77; 3.69)	2.40 (1.66; 3.47)
≥60	1.00		1.00	1.00	1.00
Tuberculosis					
No	1.00			1.00	1.00
Yes	2.85 (2.06; 3.95)			1.85 (1.29; 2.66)	1.76 (1.22; 2.54)
**Street dweller**					
No	1.00				1.00
Yes	3.27 (2.74; 3.92)				1.75 (1.41; 2.18)

^a^Model 2: Sex and schooling; ^b^Model 3: Sex, schooling and age group; ^c^Model 4: Sex, schooling, age group and tuberculosis; ^d^Model 5: Sex, schooling, age group, tuberculosis and street dweller.

## Discussion

The study investigated the characteristics of all rapid HIV tests in Porto Alegre, exploring how the race/skin color variable may be associated with higher HIV detection. Compared to White people, we found higher HIV prevalence among Black people, especially among Black men aged 40 to 59. In addition, prevalence was higher among Black people with incomplete elementary education, diagnosed with tuberculosis, as well as street dwellers.

A limitation of the study is that the period in which the rapid tests were performed (2020 to 2022) needs to be taken into account, as it coincided that the COVID-19 pandemic, when health services had access restrictions. Another possible limitation is related to loss of information, common in studies with secondary data. Despite this, the large amount of data analyzed provided robust models and a broad view of the tests performed in the city, as it covers data on rapid tests performed in Porto Alegre during the period studied.

Disparities in HIV indicators between Black and White people, such as higher detection and mortality rates (5), reveal significant health inequalities. This study identified higher HIV prevalence among Black people compared to White people. This inequality is related to a complex intersection of social, economic, and structural factors that increase and perpetuate vulnerability to HIV infection (14-15). In addition to race/skin color, other sociodemographic factors also impact vulnerability and higher HIV prevalence.

In terms of gender, men account for the majority of HIV-positive cases, which can be attributed to behavioral and social factors. These factors include higher frequency of unprotected sexual intercourse, greater consumption of psychoactive substances, and alcohol use. In addition, limited access to sexual education and health services, as well as stigmas regarding HIV, also contribute to higher prevalence among this group (16,17).

When assessing age range, it can be seen that the majority of Black individuals with a reactive HIV result were between 40 and 59 years old. Similarly, other studies have identified that HIV infection prevalence was higher among young and middle-aged people, associated with a greater number of partners and greater life-long sexual exposure (18-20).

The high HIV detection rate among Black individuals with low levels of education may be related to less knowledge about STI transmission and diagnosis (9). Individuals with lower levels of education are more likely to have an early sexual debut and to use condoms less regularly (21). In the Brazilian educational context, there is a higher dropout rate among Black people and among males (22). In addition to their level of education, HIV prevalence rates among street dwellers can reach up to 21% (23). The high number of positive rapid HIV test results recorded in this group highlights this condition of extreme vulnerability, especially considering street dwellers of Black and mixed race/skin color. Furthermore, these individuals are vulnerable to tuberculosis infection due to poor housing conditions and limited access to health services, but also due to a complex intersection of socioeconomic and racial factors that contribute to this reality. In Brazil, the overall prevalence of tuberculosis and HIV co-infection was 9.3% in 2023, and it is believed that these numbers could be even higher, given the difficulty of diagnosis among vulnerable populations (24,25).

Considering testing availability in primary health care services, knowledge of the characteristics of patients tested is essential for guiding policies and aligning strategies with local reality. In this sense, the results of this study show that there is a higher HIV detection rate among Black people in Porto Alegre. In addition, some sociodemographic and clinical characteristics further accentuate this vulnerability to HIV among Black people, such as being male, having a low level of education, having HIV and tuberculosis co-infection, and being a street dweller. As such, it is crucial that inclusive and focused strategies be formulated and implemented for this population, with the aim of reducing health inequities, addressing the HIV epidemic as one that is directly impacted by social determinants.

## Data Availability

Public access to the database used in this study is not available.
