# Supplementary material for: HIV prevalence and factors associated with HIV positivity among Black people in primary care in Porto Alegre, Brazil, 2020-2022: a cross-sectional study
Source: Epidemiol Serv Saude. 2025 May 12;34:e20240014. doi: 10.1590/S2237-96222025v34e20240014.en (PMC12077543; doi:10.1590/S2237-96222025v34e20240014.en)
Supplement: Translation

# Translation: Prevalência de HIV e fatores associados à positividade entre pessoas negras na atenção primária de Porto Alegre, 2020-2022: estudo transversal

**DOI:** 10.1590/S2237-96222025v34e20240014.pt

**Authors:** Emerson Silveira de Brito, Marsam Alves de Teixeira, Rafael Steffens Martins, Ben Hur Graboski Pinheiro, Ana Carolina Monteiro da Rocha, Cáren Nunes de Oliveira, Thayane Fraga de Paula, Thayane Martins Dornelles

**Affiliations:** 1 Universidade Federal de Ciências da Saúde, Programa de Pós-graduação em Ciências da Saúde, Porto Alegre, RS, Brasil; 2 Afya Educação Médica, Diretoria de Pós-Graduação Médica, Porto Alegre, RS, Brasil; 3 Associação Hospitalar Moinhos de Vento, Porto Alegre, RS, Brasil; 4 Instituto René Rachou, Fiocruz Minas Gerais, Belo Horizonte, MG, Brasil

**Keywords:** Infecções Sexualmente Transmissíveis, Infecções por HIV, População Negra, Saúde de Minorias Étnicas, Estudos Transversais

## Abstract

**Objetivo:**

Verificar a prevalência de HIV em usuários da atenção primária e investigar fatores associados ao teste de HIV positivo em pessoas negras.

**Métodos:**

Estudo transversal com dados de testagem rápida para HIV em unidades de saúde de Porto Alegre. Diferenças sociodemográficas de acordo com raça/cor de pele e resultado do teste de HIV foram analisadas usando teste de Qui-quadrado e regressão de Poisson com variância robusta.

**Resultados:**

Entre 92.345 pessoas testadas, 38% eram negras, com prevalência de 3,4% de HIV. Entre a população negra, esteve associado à maior razão de prevalência (RP) para HIV: ser homem (RP 1,62; intervalo de confiança de 95% (IC95%) 1,41; 1,85), possuir ensino fundamental (RP 1,69; IC95% 1,27; 2,24), ter tuberculose (RP 1,76; IC95% 1,22; 2,54) e estar em situação de rua (RP 1,75; IC95% 1,41; 2,18).

**Conclusão:**

A população negra, especialmente homens negros com menor escolaridade, tuberculose e em situação de rua, apresentam maior prevalência de HIV, exigindo maior atenção das estratégias de prevenção e testagem para infecções sexualmente transmissíveis.

## Introdução

O vírus da imunodeficiência humana (HIV) se constitui em um grande desafio de saúde pública, devido à sua magnitude, complexidade e dificuldade de controle na sociedade (1). Estima-se que 39 milhões de indivíduos estejam infectados mundialmente com o vírus, e, mesmo com os avanços no manejo da infecção, esta doença se mantém como um problema latente, sobretudo ao se avaliarem diferenças desproporcionais na prevalência do HIV em populações de maior vulnerabilidade, como pessoas negras (2-4).

No Brasil, foram notificados 37.023 novos casos de infecção pelo HIV em 2020 e 43.403 em 2022, representando um aumento de 17,2% (5). Avaliando esses novos diagnósticos de acordo com a raça/cor da pele, dados do Ministério da Saúde apontam para um aumento no diagnóstico de pessoas pardas e pretas e uma diminuição nos novos casos em pessoas brancas. Em 2023, os negros representavam 61,6% dos novos casos (49,3% pardos e 12,2% pretos), enquanto os brancos correspondiam a 32,9% (5-6). No mesmo ano, a população negra representou 63% dos óbitos em decorrência da aids no Brasil (5). Dados de outros países, como os Estados Unidos, mostram que 31% de todos os casos de clamídia, gonorreia e sífilis ocorreram entre a população negra em 2021, embora representassem aproximadamente 12% da população do país (6).

Para compreender essas iniquidades, estudos evidenciam que a população negra sofre maiores restrições de acesso aos serviços de saúde, e, quando os negros são atendidos, recebem um atendimento de pior qualidade e menor resolutividade (7). Se comparadas com a população branca, as desigualdades sociais enfrentadas por pessoas pretas e pardas resultam em maior incidência de doenças determinadas socialmente, além de maiores taxas de mortalidade, decorrentes da negligência das políticas de saúde (8-9), o que pode ser explicado por um complexo conjunto de diferentes tipos de vulnerabilidades (sociais, individuais e estruturais) determinados historicamente (10-11). 

Diante desse cenário epidemiológico entre pessoas negras, torna-se crucial a produção de novas estimativas regionais de prevalência de HIV e a análise aprofundada dos fatores associados à maior detecção do vírus nessa população. Essas iniciativas são fundamentais para fortalecer políticas de saúde e desenvolver estratégias específicas voltadas aos grupos de maior vulnerabilidade. Neste contexto, o objetivo deste estudo é verificar a prevalência de HIV em usuários da atenção primária e investigar fatores associados ao teste de HIV positivo em pessoas negras.

## Métodos

### Delineamento do estudo

Trata-se de estudo transversal com pessoas com 12 anos ou mais que realizaram testes rápidos de HIV entre janeiro de 2020 e novembro de 2022 em serviços de atenção primária de Porto Alegre. As diretrizes do *Strengthening the Reporting of Observational Studies in Epidemiology* orientaram a redação deste estudo (12).

### Participantes

Foram incluídos no estudo todos os indivíduos maiores de 12 anos de idade. Indivíduos que não apresentavam data de nascimento foram excluídos do banco. Os dados do estudo referem-se aos indivíduos residentes do município de Porto Alegre, que realizaram teste rápido para HIV nas instituições de saúde da cidade, como unidades de saúde da atenção primária do município, além dos seguintes serviços especializados: Serviços de Atendimento Especializado, Centro de Referência em Tratamento da Tuberculose e Centro de Atenção Psicossocial.

### Variáveis

A partir dos dados coletados via Google Forms pelos serviços de saúde, foram utilizadas as seguintes variáveis: raça/cor da pele (branco, negro [preto e pardo], amarelo e indígena), sexo (masculino, feminino), idade (em anos: 12-17, 18-25, 26-39, 40-59) ano da testagem rápida, escolaridade (até ensino fundamental, médio, superior) tuberculose (não, sim), situação de rua (não, sim). Também foi coletado o resultado do primeiro teste de HIV (não reagente, reagente e não realizado), seguido pelo teste confirmatório para HIV nos casos em que o primeiro teste foi positivo. Dados incompletos devido à inexistência de resposta e/ou resposta inválida foram considerados perdas.

### Fonte de dados/mensuração

O estudo foi realizado com dados secundários obtidos através da Coordenação de Atenção às Infecções Sexualmente Transmissíveis de Porto Alegre. O registro das informações sobre cada participante ocorreu a partir do preenchimento de um formulário via Google Forms por profissionais que realizam os testes rápidos para infecção sexualmente transmissível (IST), sendo esse procedimento padrão nos serviços que realizam a testagem. A gestão e a elaboração das variáveis desse formulário são realizadas pela equipe técnica de IST do município. Os dados foram acessados a partir de janeiro de 2023 após terem sido disponibilizados pela Coordenação de Atenção às Infecções Sexualmente Transmissíveis de Porto Alegre, onde estavam compilados em uma planilha do Excel contendo informações provenientes dos serviços de saúde do município.

### Tamanho do estudo

O estudo foi composto por um total de 99.641 participantes que realizaram teste rápido para HIV em Porto Alegre. Não foi realizado cálculo amostral, pois o estudo foi desenvolvido com todos os dados de testagem rápida realizados entre janeiro de 2020 e novembro de 2022.

### Métodos estatísticos

Os dados foram compilados em planilhas de Excel e posteriormente codificados para análise quantitativa no software Statistical Package for the Social Sciences (SPSS), versão 29. 

Foi apresentado o perfil sociodemográfico dos indivíduos que realizaram a testagem rápida para o HIV, discriminados por raça/cor da pele. Em seguida, apresentou-se a comparação entre perfil sociodemográfico de acordo com a positividade do teste rápido para HIV entre as populações negra e branca. Para ambas as análises, apresentadas nas Tabelas 1 e 2, as variáveis categóricas foram sumarizadas por meio de frequências absolutas e porcentagens, e avaliou-se diferenças entre os grupos utilizando-se o teste qui-quadrado de Pearson.

Além disso, foi realizada análise univariada de regressão de Poisson com variância robusta, de todas as variáveis de interesse (modelo 1). Para a análise multivariada, um quadro teórico foi estruturado de forma escalonada, com base no modelo hierárquico, para calcular as associações entre o resultado positivo para HIV (desfecho do modelo) e variáveis sociodemográficas, buscando identificar, especificamente entre a população negra, quais características poderiam estar independentemente associadas à maior prevalência de HIV. Nesse sentido, foram realizados os seguintes modelos: modelo 1 – estimativas brutas (modelo univariado); modelo 2 – sexo e escolaridade (ensino fundamental, médio ou superior); modelo 3 – sexo, escolaridade e faixa etária no momento da testagem (12-17; 18-25; 26-39; 40-59 anos); modelo 4 – sexo, escolaridade, faixa etária e histórico de tuberculose (sim/não); e modelo 5 – sexo, escolaridade, faixa etária, histórico de tuberculose e situação de rua (sim/não).

## Resultados

Foram analisados um total de 99.641 testes rápidos para IST (sífilis, HIV, hepatite B ou C) no período avaliado, sendo que 1.915 testes foram excluídos das análises devido à falta de informação de data de nascimento. Especificamente para HIV, 92.345 testagens foram realizadas e 2.319 tiveram resultado reagente, resultando em uma prevalência de 2,5% e intervalo de confiança de 95% (IC95% 2,4; 2,6). A média e desvio-padrão da idade dos participantes foi de 32,7±13,2 anos. A Tabela 1 apresenta as características sociodemográficas dos indivíduos que realizaram a testagem rápida nos serviços públicos de saúde, mostrando que, entre os indivíduos com curso superior, 73,8% eram brancos e 25,4% eram negros. Em relação aos diagnósticos de HIV, 52,4% dos testes reagentes foram em indivíduos negros. 

**Tabela 1:** Características dos indivíduos que realizaram teste rápido para HIV de acordo com a classificação de raça/cor da pele. Porto Alegre, 2020-2022 (n=92.345)

Características	Total	Brancos N (%)	Negros N (%)	Amarelos/indígenas N (%)	p-valor^a^
**Sexo**					<0,001
Homens	34.849	20.614 (59,2)	13.854 (39,7)	381 (1,1)	
Mulheres	57.496	35.231 (61,3)	21.713 (37,7)	552 (1,0)	
**Faixa etária** (anos)					
12-17	4.261	2.406 (56,5)	1.795 (42,1)	60 (1,4)	<0,001
18-25	29.023	16.914 (58,3)	11.817 (40,7)	292 (1,0)	
26-39	35.105	21.112 (60,1)	13.661 (39,0)	332 (0,9)	
40-59	17.407	10.955 (62,9)	6.260 (36,0)	192 (1,1)	
≥60	4.791	3.403 (71,0)	1.352 (28,2)	36 (0,8)	
**Escolaridade**					<0,001
Fundamental	27.767	14.960 (52,9)	12.710 (45,8)	383 (1,3)	
Médio	35.710	22.055 (61,8)	13.363 (37,4)	298 (0,8)	
Superior	8.068	5.949 (73,8)	2.053 (25,4)	69 (0,8)	
Tuberculose					<0,001
Não	75.541	45.195 (59,9)	29.573 (39,1)	773 (1,0)	
Sim	725	381 (52,6)	338 (46,6)	6 (0,8)	
**Situação de rua**					<0,001
Não	74.022	44.479 (60,1)	28.801 (38,9)	742 (1,0)	
Sim	2.244	1.097 (48,9)	1.110 (49,5)	37 (1,6)	
**Resultado TR HIV**					<0,001
Não reagente	90.026	54.765 (60,8)	34.353 (38,2)	908 (1,0)	
Reagente	2.319	1.080 (46,5)	1.214 (52,4)	25 (1,1)	

Considerando-se que os dados são provenientes de um questionário secundário, houve perdas em algumas variáveis, com as seguintes proporções de respostas válidas: sexo do paciente (98,7%), idade (96,8%), escolaridade (76,4%), situação de rua (81,5%), tuberculose (81,5%) e raça/cor da pele (98,7%). Essas variáveis foram impactadas por falhas no preenchimento ou alterações no questionário ao longo do tempo.

A Tabela 2 mostra que a prevalência de HIV entre os homens negros foi de 4,7%, e entre homens brancos, de 2,9%. Entre as mulheres autodeclaradas brancas e negras a prevalência de HIV foi de 1,4% e 2,6%, respectivamente. Considerando-se a população negra, identificou-se maior proporção de testes reagentes para HIV em pessoas de 40 a 59 anos (6,3%), com ensino fundamental (4,2%), com tuberculose (10,1%) e em situação de rua (11,1%). Quando analisamos estas mesmas variáveis para a população branca, identificamos uma maior prevalência de testes rápidos positivos entre indivíduos de 40 a 59 anos (3,0%), com ensino fundamental (2,8%), com tuberculose (7,3%) e situação de rua (6,6%).

**Tabela 2:** Características dos indivíduos e positividade do teste rápido para HIV de acordo com raça/cor da pele. Porto Alegre, 2020-2022 (n=91.412)

	Negros - n (%)			Brancos - n (%)		
Características	Não reagente	Reagente	p-valor^a^	Não reagente	Reagente	p-valor^a^
Sexo			<0,001			<0,001
Homens	13.204 (95,3)	650 (4,7)		20.016 (97,1)	598 (2,9)	
Mulheres	21.149 (97,4)	564 (2,6)		34.749 (98,6)	482 (1,4)	
**Faixa etária** (anos)			<0,001			<0,001
12-17	1.776 (98,9)	19 (1,1)		2.389 (99,3)	17 (0,7)	
18-25	11.599 (98,2)	218 (1,8)		16.711 (98,8)	203 (1,2)	
26-39	13.137 (96,2)	524 (3,8)		20.660 (97,9)	452 (2,1)	
40-59	5.864 (93,7)	396 (6,3)		10.627 (97,0)	328 (3,0)	
≥60	1.314 (97,2)	38 (2,8)		3.349 (98,4)	54 (1,6)	
Escolaridade			<0,001			<0,001
Fundamental	12.174 (95,8)	536 (4,2)		14.278 (97,2)	412 (2,8)	
Médio	12.976 (97,1)	387 (2,9)		21.692 (98,4)	363 (1,6)	
Superior	2.001 (97,5)	52 (2,5)		5.872 (98,7)	77 (1,3)	
**Tuberculose**			<0,001			
Não	28.570 (96,6)	1003 (3,4)		44.324 (98,1)	871 (1,9)	
Sim	304 (89,9)	34 (10,1)		353 (92,7)	28 (7,3)	
**População de rua**			<0,001			<0,001
Não	27.887 (96,8)	914 (3,2)		43.652 (98,1)	181 (1,9)	
Sim	987 (88,9)	123 (11,1)		1.025 (86,4)	72 (6,6)	

Os resultados da análise multivariada mostraram que a razão de prevalência entre pessoas negras com testes rápidos reagentes para HIV foi maior em homens (RP 1,62; IC95% 1,41; 1,85), pessoas com ensino fundamental (RP 1,69; IC95% 1,27; 2,24), pessoas com tuberculose (RP 1,76; IC95% 1,22; 2,54), e em situação de rua (RP 1,75; IC95% 1,41; 2,18) no modelo final. Destaca-se que pessoas negras de 40 a 59 anos apresentaram uma razão de prevalência maior para infecção pelo HIV (RP 2,40; IC95% 1,66; 3,47), enquanto a faixa etária entre 12 e 17 anos (RP 0,44; IC95% 0,24; 0,82) esteve associada à menor prevalência (Tabela 3).

**Tabela 3:** Análise multivariada de fatores associados aos resultados reagentes para HIV na população de raça/cor da pele negra. Porto Alegre, 2020-2022

Variável	Razão de prevalência (intervalo de confiança [IC] de 95%)				
	Modelo 1 (análise bruta)	Modelo 2^a^	Modelo 3^b^	Modelo 4^c^	Modelo 5^d^
Sexo					
Homens	1,84 (1,63; 2,09)	1,87 (1,65; 2,12)	1,74 (1,54; 1,98)	1,73 (1,52; 1,96)	1,62 (1,41; 1,85)
Mulheres	1,00	1,00	1,00	1,00	1,00
Escolaridade					
Fundamental	1,66 (1,25; 2,20)	1,69 (1,27; 2,23)	1,82 (1,37; 2,42)	1,30 (0,97; 1,74)	1,69 (1,27; 2,24)
Médio	1,15 (0,87; 1,54)	1,15 (0,87; 1,53)	1,30 (0,97; 1,74)	1,80 (1,35; 2,39)	1,28 (0,96;1,71)
Superior	1,00	1,00	1,00	1,00	1,00
**Faixa etária** (anos)					
12-17	0,38 (0,22; 0,66)		0,43 (0,23; 0,79)	0,44 (0,24; 0,81)	0,44 (0,24; 0,82)
18-25	0,67 (0,47; 0,94)		0,83 (0,57; 1,22)	0,84 (0,57; 1,23)	0,84 (0,57; 1,23)
26-39	1,38 (1,00; 1,91)		1,67 (1,17; 2,41)	1,68 (1,16; 2,42)	1,63 (1,13; 2,36)
40-59	2,24 (1,61; 3,11)		2,54 (1,77; 3,67)	2,55 (1,77; 3,69)	2,40 (1,66; 3,47)
≥60	1,00		1,00	1,00	1,00
Tuberculose					
Não	1,00			1,00	1,00
Sim	2,85 (2,06; 3,95)			1,85 (1,29; 2,66)	1,76 (1,22; 2,54)
**População de rua**					
Não	1,00				1,00
Sim	3,27 (2,74; 3,92)				1,75 (1,41; 2,18)

^a^Modelo 2: Sexo e escolaridade; ^b^Modelo 3: Sexo, escolaridade e faixa etária; ^c^Modelo 4: Sexo, escolaridade, faixa etária e tuberculose; ^d^Modelo 5: Sexo, escolaridade, faixa etária, tuberculose e situação de rua.

## Discussão

O estudo investigou as características de todos os testes rápidos para HIV em Porto Alegre, explorando como a variável raça/cor da pele pode estar associada à maior detecção de HIV. Em comparação com pessoas brancas, evidenciamos uma maior prevalência de HIV na população negra, com destaque para homens negros na faixa etária de 40 a 59 anos. Além disso, a prevalência mostrou-se mais elevada entre negros com ensino fundamental incompleto, com diagnóstico de tuberculose e em situação de rua.

Como limitação do estudo, é preciso levar em consideração o período da realização dos testes rápidos (2020 a 2022), que ocorreu durante a pandemia de covid-19, quando os serviços de saúde apresentaram restrições de acesso. Outra possível limitação está relacionada à perda de informações, comum em estudos com dados secundários. Apesar disso, o grande quantitativo de dados analisados forneceu modelos robustos e uma visão ampla das testagens realizadas na cidade, pois se trata de todos os dados de testes rápidos realizados em Porto Alegre no período estudado. 

As disparidades nos indicadores de HIV entre a população negra e a branca, como as maiores taxas de detecção e mortalidade (5), revelam uma significativa desigualdade em saúde. Neste estudo, identificou-se maior prevalência de HIV na população negra, em comparação com a população branca. Essa desigualdade está relacionada a uma complexa intersecção de fatores sociais, econômicos e estruturais que aumentam e perpetuam a vulnerabilidade à infecção pelo HIV (14-15). Além da raça/cor da pele, outros fatores sociodemográficos também impactam a vulnerabilidade e maior prevalência do HIV.

Em relação ao gênero, os homens constituem a maioria dos casos positivos para HIV, o que pode ser atribuído a fatores comportamentais e sociais. Entre esses fatores, destacam-se a maior frequência de relações sexuais desprotegidas, maior consumo de substâncias psicoativas e uso de álcool. Além disso, o acesso limitado à educação sexual e aos serviços de saúde, e estigmas em relação ao HIV, também contribuem para a prevalência mais alta entre esse grupo (16,17).

Ao avaliar a faixa etária, observa-se que a maioria dos indivíduos da população negra com resultado reagente para HIV estava entre 40 e 59 anos. Da mesma forma, outros estudos identificaram que populações jovens e de meia-idade apresentaram prevalências mais elevadas de infecção pelo HIV, associadas ao maior número de parceiros e maior exposição sexual durante a vida (18-20). 

A alta detecção de HIV em indivíduos negros com baixa escolaridade pode estar relacionada a um menor conhecimento sobre a transmissão e diagnóstico das IST (9). Indivíduos com menor escolaridade possuem maior probabilidade de iniciar a vida sexual precocemente e usar preservativo com menor regularidade (21). No contexto educacional brasileiro, observa-se uma evasão escolar maior entre a população negra e entre indivíduos do sexo masculino (22). Além da escolaridade, indivíduos em situação de rua apresentam prevalências de HIV que podem chegar até a 21% (23). O elevado quantitativo de testes rápidos positivos para HIV registrados nesse grupo evidenciam essa condição de extrema vulnerabilidade, sobretudo considerando-se pessoas em situação de rua pretas e pardas. Ainda, destaca-se a vulnerabilidade desses indivíduos à infecção por tuberculose, devido às condições precárias de moradia e pelo acesso limitado aos serviços de saúde, mas também por uma complexa intersecção de fatores socioeconômicos e raciais que contribuem para essa realidade. No Brasil, a prevalência geral de coinfecção de tuberculose e HIV foi de 9,3% em 2023, e acredita-se que esses números possam ser ainda maiores, tendo em vista a dificuldade de diagnóstico em populações vulneráveis (24,25).

Considerando-se a disponibilidade dos testes na atenção primária à saúde, o conhecimento das características dos pacientes testados é fundamental para a orientação de políticas e o alinhamento das estratégias com a realidade local. Nesse sentido, os resultados deste estudo evidenciam que a população negra apresenta maior detecção de HIV em Porto Alegre. Além disso, algumas características sociodemográficas e clínicas acentuam ainda mais essa vulnerabilidade em relação ao HIV na população negra, como ser homem, possuir baixa escolaridade, apresentar coinfecção de HIV e tuberculose, e estar em situação de rua. Nesse sentido, é crucial que sejam formuladas e implementadas estratégias inclusivas e focalizadas nessa população, com o intuito de se reduzirem as iniquidades em saúde, enfrentando-se a epidemia do HIV como uma epidemia diretamente impactada por determinantes sociais.

## Data Availability

O banco de dados utilizado neste estudo não é de acesso público.
